# Supplementary material for: MADloy: robust detection of mosaic loss of chromosome Y from genotype-array-intensity data
Source: BMC Bioinformatics. 2020 Nov 23;21:533. doi: 10.1186/s12859-020-03768-z (PMC7682048; doi:10.1186/s12859-020-03768-z)
Supplement: Supplementary file 1 — Additional file 1. Supplementary tables and figures. [file 12859_2020_3768_MOESM1_ESM.pdf]

MADloy: Robust detection of mosaic loss of chromosome Y from genotype-array-intensity data

Supplementary Material

Juan R González[1,2,3,*], Marcos López-Sánchez[4], Alejandro Cáceres[1,2],
Pere Puig[3], Tonu Esko[5], Luis A Pérez-Jurado[4,5,6]

id	mLRR	MADloy (LRR)	mLRR-Y_{thres}	MADloy (LRR + Bdev)
V17493	-0.1	normal	LOY	discordant
V25393	-0.09	normal	LOY	other
V26411	-0.11	normal	LOY	other
V26498	-0.08	normal	LOY	discordant
V38138	-0.1	normal	LOY	discordant
V38996	-0.08	normal	LOY	discordant
V39073	-0.11	normal	LOY	discordant
V39233	-0.13	normal	LOY	discordant
V40705	-0.1	normal	LOY	discordant
V45177	-0.09	normal	LOY	discordant

Table S1: Discordant calls of EGCUT data sets. Data of some one case with discordant and other classification by MADloy are visually inspected in Figure S3.

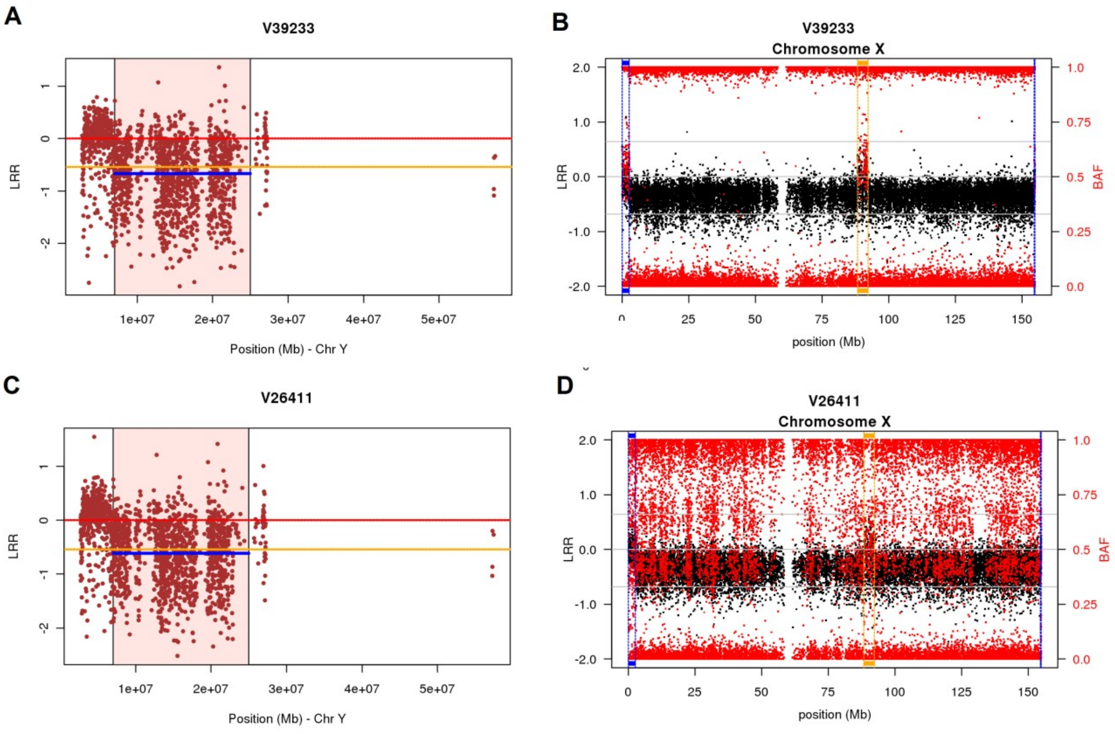

Figure S1: Panels A and C show LRR and mLRR-Y of chromosome Y. Panels B and D show LRR and BAF of chromosome X of two samples from ECGUT dataset that were called as “discordant” (V39233) and “other” (V26411)
MADloy. Sample V39233 (panels A and B) has been classified as “discordant” due to a decreased LRR (panel A, blue line) than expected (panel A, orange line) but no clear BAF split is visible in PAR1, PAR2 (panel C, Blue boxes) and XTR (panle C, orange box) in chromosome X. Sample V26411 (panels C and D) has been classified as “other” due to a decreased LRR(C, blue) than expected (panel C, orange line). However, by looking at chromosome X plot (panel D), it can be seen a 2-band BAF split caused by the presence of an XX sample contamination that will also decrease the expected chromosome Y dosage in the sample.

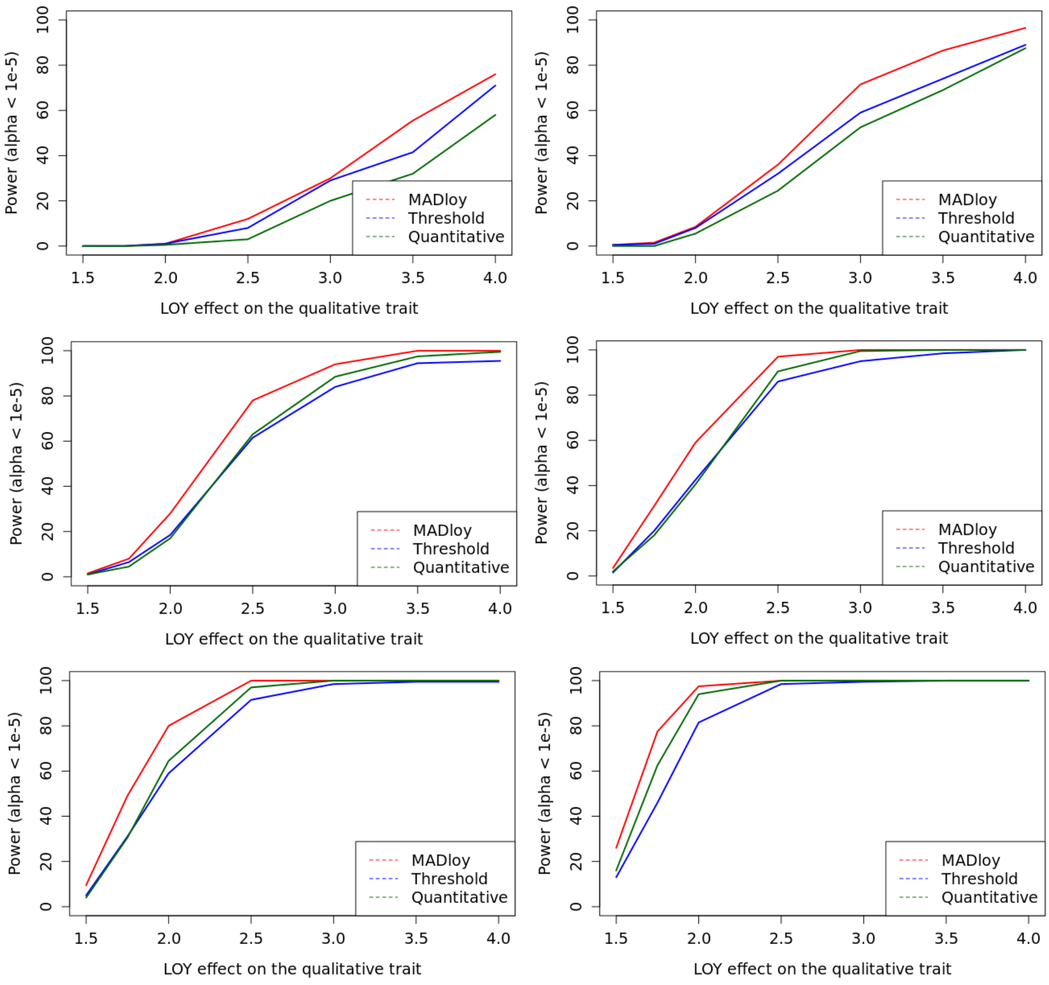

Figure S2: Simulation results for quantitative traits (e.g. age) for different methods. MADloy: uses mLRR-Y information, Threshold: mLRR-Y_{thres} (Fosberg et al., 2014), and Quantitative: mLRR-Y_{quant} (Wright et al., 2017). Each panel shows the results for different sample sizes (from left to right and top to bottom: 200, 300, 500, 750, 1000, 1500). The study assess the power (p-value<10^{-5}) to detect differences in the mean of the quantitative trait in the range 1.5 up to 4. The scenarios and how data are simulated are described in the Methods Section of the main manuscript.

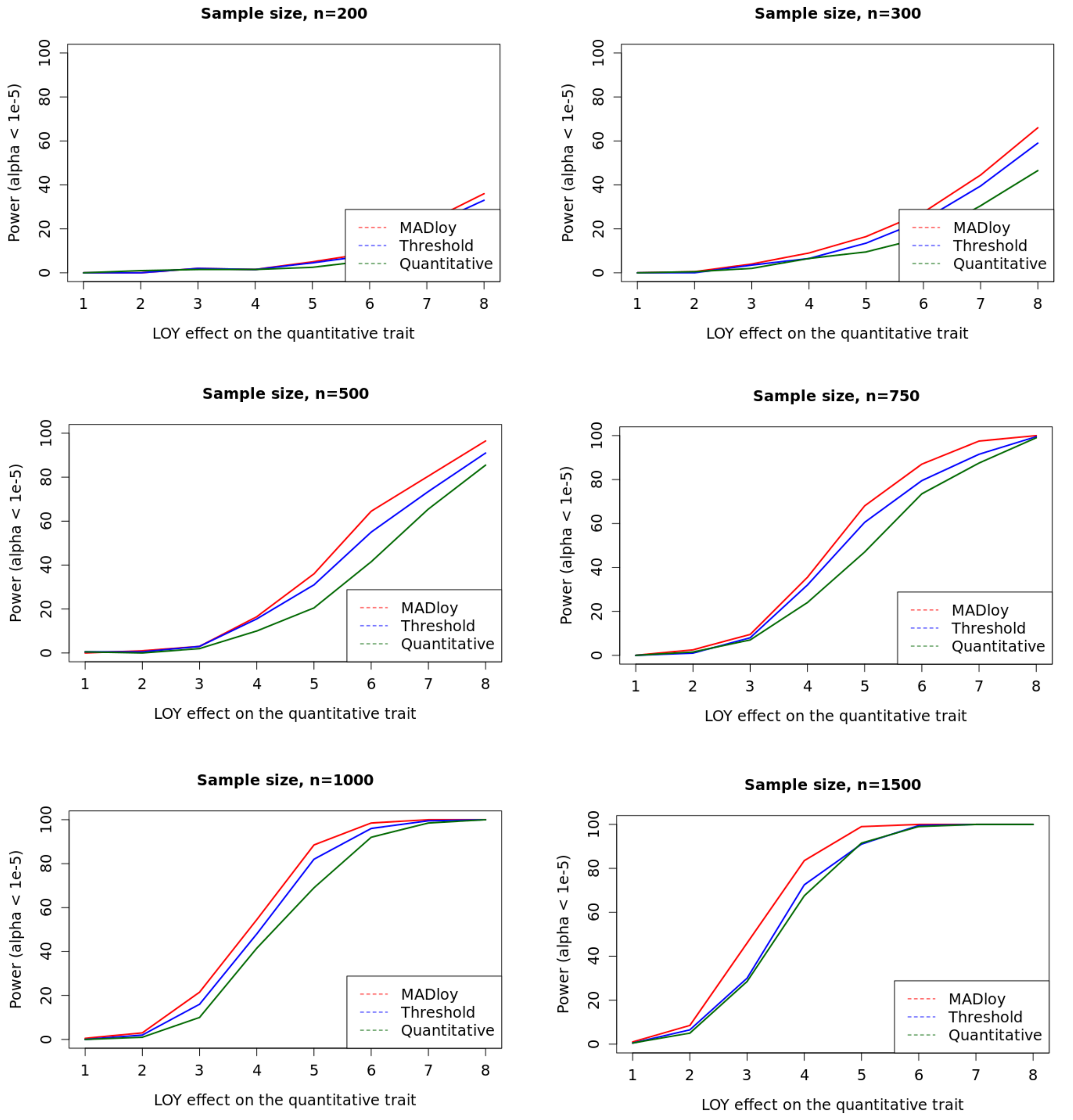

Figure S3: Simulation results for binary traits (e.g. case/control) for different methods. MADloy: uses mLRR-Y information, Threshold: mLRR-Y_{thres} (Fosberg et al., 2014), and Quantitative: mLRR-Y_{quant} (Wright et al., 2017). Each panel shows the results for different sample sizes (from left to right and top to bottom: 200, 300, 500, 750, 1000, 1500). The study assess the power (p-value<10^{-5}) to detect differences between cases an controls at different levels (odds ratios ranging from 1 up to 8). The scenarios and how data are simulated are described in the Methods Section of the main manuscript.

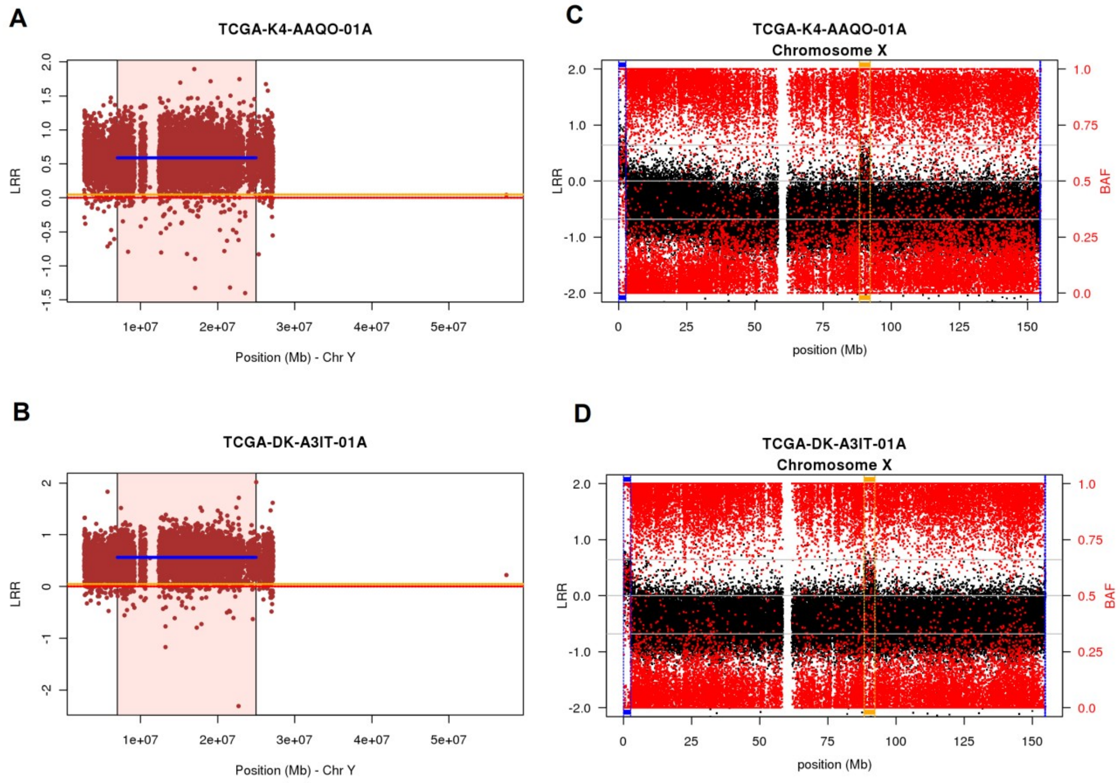

Figure S4: Panels A and C show LRR and mLRR-Y of chromosome Y. Panels B and D show LRR and BAF of chromosome X of two samples from TCGA dataset who were called as GOY. Sample TCGA-K4-AAQO-01A has an increased mLRR-Y (blue line panel A) which is far from the expected value (orange line panel A). Additionally, the LRR and BAF plot of chromosome X (panel C) shows a BAF split in the PAR1, PAR2 (blue boxes) and XTR (orange boxes), compatible with the allelic imbalance between the two Y and one X chromosome in the sample analyzed. The same is observed for sample TCGA-DK-A3IT-01A, where the mLRR-Y (panel B) and the LRR and BAF plot of chromosome X (planel D) shows the same pattern.
